# Toward A New Paradigm of Genomics Research —Celebration of the 20th Anniversary of Beijing Institute of Genomics

**DOI:** 10.1016/j.gpb.2023.10.005

**Published:** 2023-10-29

**Authors:** Zhang Zhang, Songnian Hu, Jun Yu

**Affiliations:** 1 National Genomics Data Center, Beijing Institute of Genomics, Chinese Academy of Sciences and China National Center for Bioinformation, Beijing 100101, China; 2 CAS Key Laboratory of Genome Sciences and Information, Beijing Institute of Genomics, Chinese Academy of Sciences and China National Center for Bioinformation, Beijing 100101, China; 3 University of Chinese Academy of Sciences, Beijing 100049, China; 4 State Key Laboratory of Microbial Resources, Institute of Microbiology, Chinese Academy of Sciences, Beijing 100101, China

**Keywords:** Genomics, Bioinformatics, BIG, CNCB, NGDC

## Abstract

Twenty years after the completion and forty years after the proposal of the Human Genome Project (HGP), genomics, together with its twin field — bioinformatics, has entered a new paradigm, where its bioscience-related, discipline-centric applications have been creating many new research frontiers. Beijing Institute of Genomics (BIG), now also known as China National Center for Bioinformation (CNCB), will play key roles in supporting and participating in these frontier research activities. On the 20th anniversary of the establishment of BIG, we provide a brief retrospective of its historic events and ascertain strategic research directions with a broader vision for future genomics, where digital genome, digital medicine, and digital health are so structured to meet the needs of human life and healthcare, as well as their related metaverses.

Along with the initiation, execution, and accomplishment of the Human Genome Project (HGP), genomics, an interdisciplinary field studying organismal genome and its structure in a context of evolutionary changes among lineages, has been developed at an extraordinary pace in the past decades, exerting an indispensable and catalytic role in a wide range of life, medicine, and health sciences. Aside from having fulfilled its goals as a multi-national research project, HGP has expedited the emergence of institutions worldwide and among them are the Beijing Institute of Genomics (BIG), founded officially within the Chinese Academy of Sciences (CAS) in 2003 in China, the National Human Genome Research Institute (previously the National Center for Human Genome Research) within the National Institutes of Health in 1997 in the United States (US), and the Wellcome Sanger Institute (previously the Sanger Centre) in the United Kingdom (UK) in 1993. To commemorate the 20th anniversary of the foundation of BIG, here we provide a brief retrospective of its historic events and articulate a broader vision for research directions and challenges of future genomics.

## Retrospective of BIG achievements on the 20th Anniversary

### The birth of BIG and participation in the HGP (1998–2003)

**Figure 1:**
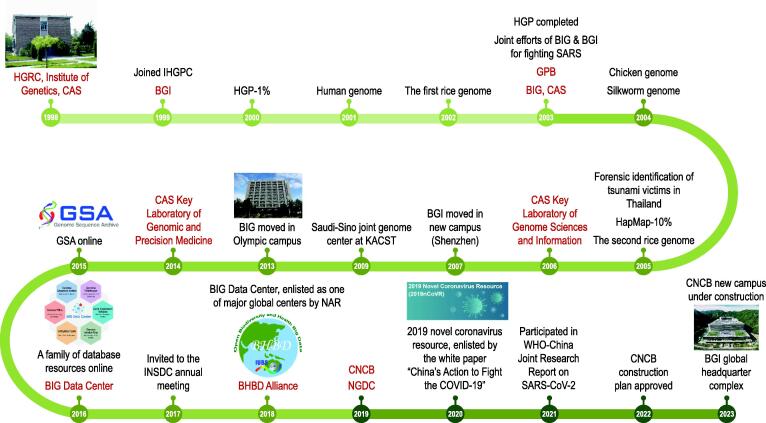
**Timeline of major events and institutionsfounded since 1998** The history of BIG roughly falls into three stages, namely, 1998–2003, 2003–2019, and 2019–now, which are marked by light green, green, and dark green, respectively, with major events (black) and institutions (red) labeled. Courtesy of Aoruan Mo for the image of BGI global headquarter complex. BGI, Beijing Genomics Institute; BHBD, Biodiversity and Health Big Data; BIG, Beijing Institute of Genomics; CAS, Chinese Academy of Sciences; CNCB, China National Center for Bioinformation; COVID-19, coronavirus disease 2019; GPB, *Genomics, Proteomics & Bioinformatics*; GSA, Genome Sequence Archive; HapMap, International HapMap Project; HGP, Human Genome Project; HGRC, Human Genome Research Center; IHGPC, International Human Genome Sequencing Consortium; INSDC, International Nucleotide Sequence Database Collaboration; KACST, King Abdulaziz City for Science and Technology; NAR, *Nucleic Acids Research*; NGDC, National Genomics Data Center; SARS-CoV-2, severe acute respiratory syndrome coronavirus 2.

The birth of BIG can be dated back to a historic conference organized by the Subcommittee of Young Geneticists of the Genetics Society of China (GSC) held in Zhangjiajie, Hunan Province, China in November of 1997, when Huanming Yang (杨焕明), Jun Yu (于军), and Jian Wang (汪建), as well as other young scientists, met together and discussed China’s possible roles in joining the HGP and engagement in genomics research (Figure 1). A year later, on August 11, 1998, the Human Genome Research Center (HGRC), BIG’s predecessor, was timely established at the Institute of Genetics (now the Institute of Genetics and Developmental Biology), CAS. On July 7, 1999, HGRC, on behalf of the Chinese scientists, made a proposal to join the international HGP Sequencing Consortium. It was officially approved on September 1, 1999 by the Consortium, so that China became the sixth member nation after the US, UK, Japan, Germany, and France, together with an assigned sequencing task that was proposed by the Chinese researchers with a claim about ∼ 30 Mb region on the short arm of human chromosome 3 as a candidate region for esophageal cancer predisposition. This task was known as the Beijing Task of HGP or the 1% Project. To secure success, Beijing Genomics Institute (BGI; https://www.genomics.cn), a private enterprise jointly founded with the help of HGRC, was established on September 9, 1999 in the Airport Industrial Zone, Shunyi District, Beijing, China. In April 2000, the 1% Project was announced its conclusion and the draft human genome was announced to be accomplished in April 2001 [1]. Given the importance of this newly-ermerged field, on November 28, 2003, BIG (http://www.big.ac.cn) was officially founded as a new institution in CAS after a 3-year development period, and its faculty and staff were mostly from HGRC and BGI. At that time, the two institutions, BIG and BGI, not only shared the same roof but also some of their personnel. Together with the launch of BIG, *Genomics, Proteomics & Bioinformatics* (GPB), a journal administrated by both BIG and GSC, has started its voyage toward future genomics, together with field pioneers, rookies, and veterans alike.

### The rapid development of genomics in China (2003–2019)

Since its establishment, BIG has been focused on genome structure, variation, function, and regulation of human and other species, contributing substantially to the rapid development of genomics research in China (Figure 1). Considering the national strategic demands coupled with the advances in biotechnologies, BIG has formed three major research units — CAS Key Laboratory of Genome Sciences & Information (CAS-KLGSI) established in 2006, CAS Key Laboratory of Genomic and Precision Medicine in 2014, and BIG Data Center [2] in 2016, where the latter two units were derived from CAS-KLGSI as its faculty’s research interests are broadening and evolving. Given the increasing significance of multi-omics data in life, medicine, and health sciences, the National Genomics Data Center (NGDC; https://ngdc.cncb.ac.cn) was founded on the basis of the BIG Data Center in June 2019, as ratified by the Ministry of Science and Technology and the Ministry of Finance of China, with joint efforts from BIG and two additional CAS institutions, *viz.*, Institute of Biophysics and Shanghai Institute of Nutrition and Health, as well as other worldwide partnerships. Shortly, on November 2019, the China National Center for Bioinformation (CNCB; https://www.cncb.ac.cn) was endorsed by the State Department to be co-administrated by BIG, which is certainly attributable to the foundation laid by CAS-KLGSI’s decade-long efforts in leading the field in China, as well as the whole scientific community in China, who advocated the establishment of CNCB since the early 1990s. The long-term goals of CNCB are to achieve centralized data deposition, security management, and public sharing of multi-omics data, support interdisciplinary cutting-edge research, and translate big data into big discoveries.

In 2007, BIG and BGI had moved out from the original joint campus at the Shunyi District and gone on their new voyages separately; the former started its building project in the CAS Olympic Village Science Park Campus, and the latter headed to a center stage of the national economic development — the Shenzhen Special Economic Zone. In seeking stronger local governmental and institutional supports for new progresses, both BIG and BGI have now well established in their premeditated physical locations; the former enjoys the close proximity with other CAS institutions by moving to the Olympic Village Science Park Campus in 2013, and the latter proceedes without hesitation “to sow more seeds in the fertile land of business and innovation”, with the global headquarter complex completed in June 2023. Despite the time interruption, a series of major breakthroughs have been achieved by both institutions in many research frontiers of genomics and bioinformatics. In particular, BIG and BGI have jointly launched several national research projects with notable success, such as the Chinese Super-hybrid Rice Genome Project [3], the Chicken Genome Project [4], the Silkworm Genome Project [5], participated in the International HapMap Project [6], and provided valuable scientific support for forensic identification of tsunami victims in Thailand [7]. Strikingly, in the past pandemics, BIG devoted timely, considerable, and vital efforts on studying the genomes of severe acute respiratory syndrome (SARS)-associated coronavirus (SARS-CoV) in 2003 [8] and SARS coronavirus 2 (SARS-CoV-2) in 2019 [9], as exemplified by building the 2019 novel coronavirus resource [10] that incorporates SARS-CoV-2 genome sequences, variants, and haplotypes based on large-scale integrative analysis of massive viral sequences worldwide. In recent years, CNCB-NGDC provides a family of database resources for big data deposition, integration, and translation [11], as typified by the Genome Sequence Archive (GSA) [12], [13], a data repository for archiving raw sequence reads that houses more than 34 perabytes (PB) of user-submitted data till October 2023.

In the course of carrying on these research activities, BIG has established broader international collaborations with different overseas institutions. Notably, a Saudi-Sino joint genome center was established in 2009 through collaboration with King Abdulaziz City for Science and Technology in Riyadh, Saudi Arabia, leading to the accomplishment of the date palm genome project [14]. Meanwhile, given the rapid accumulation of multi-omics data powered by higher-throughput sequencing technologies, the Global Biodiversity and Health Big Data (BHBD) Alliance (http://bhbd-alliance.org) was founded in 2018 under the framework of “Open Biodiversity and Health Big Data Initiative” by the International Union of Biological Sciences. With the shared aim to promote data sharing, 28 institutional members from 12 nations (as of October 2023) have joined the BHBD Alliance, leading to fruitful outcomes over the past several years, including not only data sharing across the countries but also joint-funded projects, collaborative research publications, academic conferences, and technical training programs. Moreover, owing to the recognition as one of the major global centers, CNCB-NGDC has been invited since 2017, with the purpose to establish collaborations in data sharing and exchange at a global scale, to join the annual meetings of the International Nucleotide Sequence Database Collaboration (INSDC), initiated jointly by the US National Center for Biotechnology Information (NCBI), the European Bioinformatics Institute (EBI), and the DNA Data Bank (DDBJ) of Japan.

### The foundation of CNCB and NGDC (2019–now)

Since the foundation of CNCB and NGDC in 2019, BIG has entered into a completely new stage with major efforts focusing on data resources — fundamentals in bioinformatics, yet with little attention devoted before 2019 (Figure 1). As data resources are of great significance for both academic research and national strategic needs, NGDC is the core unit of CNCB. Certainly, aside from NGDC, CNCB is going to expand gradually and set up other units, such as computational methods & algorithms, advanced biotechnologies, health & medicine, and data security, together exerting indispensable contributions in support of scientific research activities domestically and internationally. One representative example of CNCB-NGDC contributions, as mentioned above, is the 2019 novel coronavirus resource, which was enlisted by the white paper “China’s Action to Fight the COVID-19” in 2020 (http://english.scio.gov.cn/whitepapers/2020-06/07/content_76135269.htm). Furthermore, based on this resource, a series of critically fundamental research outcomes and findings were provided in aid of the WHO-China Joint Research Report on SARS-CoV-2.

The latest progress of CNCB is its construction plan, which was officially approved by the National Development and Reform Commission, China in 2022, and its new campus has been assigned in Zhangjiakou, Hebei Province, located ∼ 190 km northwest of Beijing. Collectively, this recent initiative provides great opportunities in accelerating the development of genomics and bioinformatics, driving significant scientific innovations, and potentially exerting substantial influences on national scientific research in the following years.

## Strategic future directions with enormous challenges ahead

**Figure 2:**
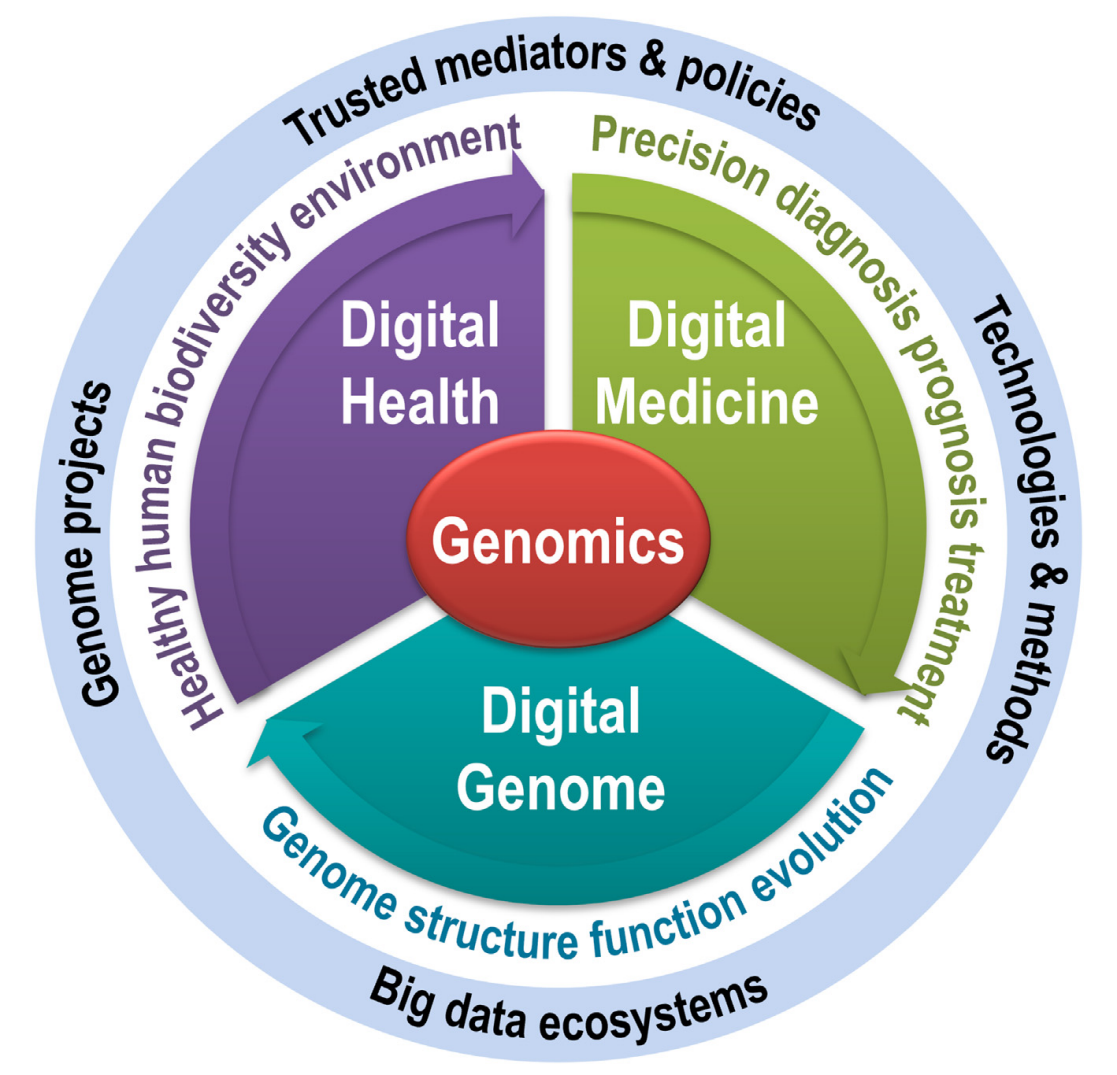
**Three-area strategic framework of genomics** The three areas are color-coded, along with their critical cross-cutting commons. Digital genome is to understand genome structure, function, evolution, *etc*.; digital medicine is for precision diagnosis, prognosis, and treatment; and digital health is to achieve one health for human, biodiversity, and environment. Cross-cutting commons include the launch of genome projects, innovation of technologies and methods, establishment of big data ecosystems, and development of trusted mediators and policies.

As genomics and bioinformatics are twin or sister disciplines and have been growing rapidly in China, it is thus easy to understand that BIG and CNCB are working together with different focuses and emphases. Considering the long-standing missions of BIG and CNCB under this situation, future strategic directions toward a broader vision for new innovations are formulated into a new paradigm [15], where the application of genomic knowledge and principles to specific fields becomes obvious [16], namely, human-centric 3D — digital genome, digital medicine, and digital health (Figure 2). Digital genome is to elucidate fundamental characteristics of genome and gene organization, structure–function relationship, genome variation, and evolution among mammals and other vertebrate lineages, leading to the understanding of genotype–phenotype associations and gene functions in both precision and thoroughness. Digital medicine, with the goal of transforming genome information into precision diagnosis, prognosis, and treatment with improved outcomes, aims to identify disease-related and/or drug-response genetic materials and develop genomics-based medical strategies in a personalized manner based on individual genetic, environmental, social, and lifestyle factors [17]. Digital health, based on the concept of one health — healthy human, biodiversity, and environment — that recognizes the interconnection among humans, animals, plants, microbes, and their shared environments, is to translate genomic information into health benefits. As the health status of our humans is related to complex interactions with other species and environments, optimal health outcomes can be achieved only when public health, health care, and disease prevention are genomically redefined from the systematic perspective.

Of note, to implement the aforementioned three-area strategic framework, it cannot be accomplished without full considerations of several critically cross-cutting commons, including genome projects, new technologies & methods, big data ecosystems, and trusted mediators & policies [17] (Figure 2). First, genome projects are crucial to decoding digital sequences for diverse species and populations at genome and pan-genome scales, often with large-scale collaborative efforts across countries and disciplines, like HGP. Yet, genome sequences alone are not sufficient; a bulk of multi-omics data are also needed for systematically determining functional elements within genomes and yielding high-quality annotations for public use. Second, technologies & methods drive significant and even paradigm-shifting innovations. Thus, development of new or improved technologies & methods is critical for generating data with higher quality and lower cost, yielding new types of data previously unreachable, and achieving data analysis & mining with higher accuracy and effectiveness, which together could potentially lead to new discoveries and new theories. Third, big data ecosystems cover the full spectrum of data cycle, involving not only infrastructure (hardware and software) but also a wide range of data resources, web systems for data, information, and knowledge, data standards from deposition to integration to translation, matched tools and interfaces, *etc*. Fourth, last but not least, trusted mediators & policies are helpful to realize the full benefits from genomic data, research findings, and discoveries, since the future of genomics also rests on shared consensuses among the general public, professionals, officials, and researchers. As an educated public is beneficial to recognize the value of genomics research, we envision an increasing task of BIG in promoting knowledge dissemination of genomics to the general public as well as the governmental and commercial sectors, increasing the wide awareness of significant roles of genomics and bioinformatics in life, medicine, and health sciences, advocating long-term stable funding support for genomics technologies and bioinformatics resources (*e.g.*, databases in CNCB-NGDC), fostering new-generation genomics and bioinformatics practitioners with multidisciplinary background, and formulating scientific policies that make full use of genome information in biological, health, and medical research settings in harmony with ethical, legal, and social regulations.

## Concluding thoughts

BIG’s roles in future genomics are multi-fold. Since the participation in HGP, genomics, together with bioinformatics and other related research activities, has been rapidly developed in China, laying important foundations and profound impacts for domestic academic research and industrial development. “*What's past is prologue*.” Considering the increasingly significant role of genomics in life, medicine, and health sciences, we outline strategic future directions toward a new paradigm of genomics research on the 20th anniversary of BIG, in which digital genome, digital medicine, and digital health are interconnected with cross-cutting commons required.

## Competing interests

The authors declare no competing interests.

## CRediT authorship contribution statement

**Zhang Zhang:** Conceptualization, Writing – original draft, Writing – review & editing, Project administration, Funding acquisition. **Songnian Hu:** Conceptualization, Writing – review & editing. **Jun Yu:** Conceptualization, Writing – review & editing, Project administration. All authors have read and approved the final manuscript.
